# A qualitative study of health care professionals’ views and experiences of paediatric advance care planning

**DOI:** 10.1186/s12904-018-0347-8

**Published:** 2018-07-13

**Authors:** Barbara A. Jack, Tracy K. Mitchell, Mary R. O’Brien, Sergio A. Silverio, Katherine Knighting

**Affiliations:** 1 Evidence-Based Practice Research Centre, Faculty of Health & Social Care, Edge Hill University, St Helens Road, Ormskirk, Lancashire L39 4QP UK; 2 Post-Graduate Medical Institute, Edge Hill University, St Helens Road, Ormskirk, Lancashire L39 4QP UK; 3 Faculty of Health & Social Care, St Helens Road, Ormskirk, Lancashire L39 4QP UK; 4 Research Department of Reproductive Health, Elizabeth Garrett Anderson Institute for Women’s Health, University College London, 74 Huntley Street, London, WC1E 6AU UK

**Keywords:** Advance care planning, End-of-life care, Interviews, Paediatrics, Palliative care, Qualitative research, Terminal care, Thematic analysis

## Abstract

**Background:**

Good end-of-life care planning is vital to ensure optimal care is provided for patients and their families. Two key factors are open and honest advance care planning conversations between the patient (where possible), family, and health care professionals, focusing on exploring what their future wishes are; and the development of an advance care plan document. However, in paediatric and neonatal settings, there has been little research to demonstrate how advance care planning conversations take place. This study explored health care professionals’ views and experiences of paediatric advance care planning in hospitals, community settings and hospices.

**Methods:**

A qualitative methodology was employed using purposive sampling of health care professionals involved in the end-of-life care for children aged 0–18 years known to the hospital palliative care team, and had died at least three months before, but less than 18 months prior to the study. Ethics committee approval was obtained for the study. Located in the North of England, the study involved three hospitals, a children’s hospice, and community services. Data were collected using semi-structured, digitally recorded, telephone interviews. All interviews were transcribed verbatim and subjected to thematic analysis.

**Results:**

Twenty-one health care professionals participated, including generalist paediatric staff as well as specialist palliative care staff.

Two themes were generated from the study: The timing of planning conversations, including waiting for the relationship with the family to form; the introduction of parallel planning; avoiding a crisis situation. Secondly, supporting effective conversations around advance care planning, including where to have the conversation; introducing the conversation; and how to approach the topic encompassing the value of advance care planning and documentation for families.

**Conclusion:**

The timing of when to start the advance care planning conversations remains an issue for health care professionals. The value of doing it in stages and considering the environment where the conversations are held was noted. Timely planning was seen as vital to avoid difficult conversations at a crisis point and for co-ordination of care. Good advance care planning is to provide the best person-centred care for the child and experience for the family.

## Background

Good end-of-life care planning is vital to ensure optimal care is provided for children, young people and their families which meet their wishes and choices, including their preferred place of care and death [1–3]. A key process to guide end-of-life planning, and the required conversations, is the use of an advance care plan document. There has been much debate as to what constitutes Advance Care Planning (ACP) and documentation, with different interpretations in both clinical settings and across countries [4]. A recent international, multi-disciplinary, expert consensus panel reported considerable complexity surrounding defining paediatric ACP [4]. In the UK, the National Institute for Health and Care Excellence (NICE) provides a definition of ACP for children and young people (including neonates) with life-limiting conditions as: “A formal care plan that includes details about the child or young person’s condition, decisions made with them and their parents or carers (for example about managing symptoms), and their wishes and ambitions. This plan is a core element of their palliative care.” ([3], p33).

Central to ACP, are open and honest communications between the children, family, and health care professionals; focusing on exploring what their future wishes and plans are. This is when a conversation takes place between children and their families, or significant others, and health care professionals to explore the child's and families’ understanding of the condition along with any potential treatment. Where possible it is recommended that parallel planning be introduced so that other supportive and palliative care options can be discussed alongside any curative approaches. Parallel planning is defined as “planning for life while also planning for deterioration or death allows a child’s full potential to be achieved and primes the mobilisation of services and professionals where necessary.” ([2], p13). National guidance and frameworks recommend the early involvement of palliative care teams to facilitate parallel planning, allowing parents time to consider all options for the care and death of their child, so appropriate documentation can be completed, whilst also facilitating emotional and psychological support for the parents [2, 3]. ACP also explores the values, beliefs, and wishes of the patient, where possible [1, 3]. These conversations may also lead to further discussions and documentation around advance directives [5], and lasting powers of attorney which are legislated in the United States of America (US) [6], Australia [7], the United Kingdom (UK) [8] and some parts of Europe [9, 10]. Furthermore, having timely advance care conversations and the use of an ACP document is internationally recommended for children and young people in guidelines and by key organisations [1, 2, 11, 12]; however, the guidance is vague about the specific timing of ACP.

There are limited studies exploring the impact of the ACP process and the use of ACP documentation in paediatric settings. The available evidence suggests that it has the potential to improve communication between patients and health care professionals, improve quality of life for patients and families, and reduce the use of futile treatments and hospitalisation [13]. One systematic review identified some general conclusions that the discussions around the ACP can positively influence emotions and aid communication with families [14]. A similar finding to a study by Lotz et al. in 2015, which reported the benefits of the planning process and the subsequent ACP documentation in providing the family with a sense of security and control, better quality of life, avoiding treatment deemed not to be in the child’s best interest, and respect for the patient’s autonomy [15].

Studies have reported challenges for health care professionals in the advance care planning process and a reluctance to have the conversations. This is particularly in cases where there is an uncertain prognosis with the family potentially giving up hope. Also reported is the negative impact on the parents of a patient signing an advance directive and parents not being ready to have the conversations [15–17]. In an American survey of 266 health professionals, a key issue highlighted was the timing of the conversations, with 71% of participants reporting it started too late and 60% referring to how often it occurred during an emergency, or when death was imminent, clearly impacting negatively on the planning process [16]. The timing of the conversations taking place after a crisis was also reported in a recent UK study [17]. Health care professionals involved in the evaluation of the introduction of a paediatric ACP emphasised the high-level of skills that were needed to have these conversations. Hospice-based staff reported feeling more comfortable than community specialist nurses, who referred to themselves as mainly dealing with generic community nursing issues, rather than paediatric end-of-life care [18].

There is a paucity of studies which explore the process and use of ACP in paediatrics. Yet with the advances in medicine and technology many children, and neonates with life-limiting conditions, are now surviving birth and living well into infancy. Although some children have a longer life expectancy and may live into adulthood [18], many, particularly those with complex syndromes, have shortened life expectancies [2]. The requirement to have timely conversations with parents, and the child, where possible, about treatment options and decisions of future care which may have to be faced over time could increase the understanding of the child’s prognosis and, more so, help them to prepare for the future [14].

The aim of this study was to explore health care professionals’ views and experiences of ACP and the documentation within the paediatric setting. The paper has drawn upon the Consolidated criteria for reporting qualitative research (COREQ) guidelines for reporting of qualitative studies [19].

## Research ethics

The study was reviewed and approved by a University Faculty Research Ethics Committee, National Health Service (NHS) Research Ethics Committee and all NHS organisations who were clinical sites (details of which can be found in the declarations section). All appropriate support and access to counselling was available to participants due to the sensitive nature of the study. All data were stored in accordance with NHS and University data management and storage policies.

## Methods

A qualitative methodological approach which drew upon a naturalistic interpretative design was employed for the study [20, 21]. This allowed us to gain an in-depth understanding of the health care professionals view and experiences of ACP and ACP documentation during end-of-life care. For the purpose of this study, we adopted the accepted UK definition of end-of-life care as relating to the final days, weeks, and months of life [3].

### Sampling

The study took place in the North of England and included three hospitals (a tertiary children’s hospital and two neonatal units), a children’s hospice, and community services in the surrounding regions across three counties. An initial screening was conducted to identify children who were (i) aged 0–18 years of age, who (ii) had died within the last 18 months, but it was more than three months since the death, and (iii) were known to the palliative care team of the tertiary children’s hospital or the children’s hospice. Purposive sampling was then used to identify health care professionals in the teams who had been involved in end-of-life care of these children, (i) from across the clinical sites, and (ii) from a range of the clinical and practitioner roles involved. This timeframe was to consider recall and to ensure it was not too soon after the death of the child [22].

### Recruitment

The service clinical leads (senior clinicians) reviewed their records and identified health care professionals who met the above inclusion criteria. Professionals were sent information about the study and those who were interested in taking part were invited to contact the research team.

### Data collection

Data collection was undertaken using semi-structured, digitally recorded, telephone interviews [23]. This approach is widely used in health service research as it enables busy professionals to take part in studies as well as capturing experiences from participants across wide geographical areas [20]. Interviews were conversational in style allowing participants to address themes relevant to two broad questions: (i) are there any practices professionals felt facilitated decision making or communication during the advance care planning process and preparation of an ACP document? and (ii) what are professionals’ views on the right time to start these planning conversations? This allowed us to follow appropriate avenues of inquiry opened by the participants, resulting in an in-depth, rich understanding of the participants’ experiences [23–26]. Interviews lasted between 20 and 60 min and were undertaken between January and July 2016 by two members of the research team (KK, TM). Participants declined to see a copy of the transcription for checking, but all requested to receive a summary report.

### Data analysis

Interviews were transcribed verbatim and anonymised during this process, thus removing identifying features. Thematic analysis was used to analyse the transcripts in a methodical and rigorous approach [26]. The approach followed key sequential steps to systematically organise, reduce, refine, and ultimately analyse the data [21]. The process involved reading each transcript in its entirety by one researcher (TM) with a sample checked by the second researcher (KK) ensuring anonymity, accuracy of transcription, and context. Two members of the research team (TM, KK) conducted the preliminary analysis generating initial codes and searching for themes by accumulating all the relevant data for each proposed theme [26]. A third researcher (BJ) reviewed and, where necessary, reconciled any discrepancies in proposed themes [27]. This increased the rigour and reduced the likelihood of introducing bias at the analysis stage [28]. Subsequently, meetings with the other members of the research team (MOB, SAS) enabled consensus to be reached on the themes allowing them to be named and defined [21, 28]. The final interpretative phase allowed the lessons learned from the analysis to be presented by using illustrative quotations to support each theme [26, 28].

## Results

Twenty-eight health care professionals were identified who met the inclusion criteria. However, seven declined to take part resulting in 21 participants. Please see Table 1.

**Table 1:** Professional participants

Role^a^	Organisation	Unique participant ID
Hospice Nurse	Hospice	P1
Obstetric and Gynaecology Consultant	Trust 3	P2
Hospice Nurse	Trust 3	P3
Consultant Paediatrician	Hospice	P4
Midwife	Trust 3	P5
Community Midwife	Hospice	P6
Neonatal Nurse	Trust 3	P7
Consultant Paediatric Oncologist	Trust 1	P8
Complimentary Therapist	Hospice	P9
Hospice Nurse	Hospice	P10
Paediatric Palliative Care Nurse	Trust 4	P11
Bereavement Specialist	Trust 1	P12
Senior Hospice Nurse	Hospice	P13
Practitioner	Hospice	P14
Health Visitor	Trust 5	P15
Care Assistant	Hospice	P16
Support Worker	Hospice	P17
Consultant Neonatologist	Trust 2	P18
Palliative Care Nurse Specialist	Trust 1	P19
Neonatal Nurse	Trust 2	P20
Hospice Nurse	Hospice	P21

^a^Due to the small and specialised nature of the paediatric end-of-life care field, broad definitions for the participants’ roles were adopted to protect each participant’s anonymity

The majority of the participants were very familiar with ACP and the documentation, due to the clinical setting they were based in, which regularly cared for children who were receiving care from the palliative care team. Only two participants reported not being familiar with ACP or the documentation; this was mainly due to their roles, which were part of additional services based in the community or in hospital teams where ACP would not be seen as a routine aspect of their role.

Two overarching themes relating to the health care professionals’ experiences of the ACP process and documentation were generated from the study. Theme one was the timing of the conversations, which included influencing factors of: point of diagnosis; waiting for the relationship with the family to form; parallel planning; and avoiding a crisis situation. The second theme was supporting effective conversations around advance care planning, with influencing factors of: where to have the conversation; flexible planning; and introducing the conversation and the value of ACP and documentation for families. Quotations from the participants are presented using best exemplars to illustrate the themes.

### The timing of the conversations

#### Waiting for the relationship with the family to form

There were differing views between the participants as to when would be the ideal time to start to have the conversations around ACP, with some professionals suggesting it should be after the relationship with the family is formed. Health care professionals repeatedly spoke about how the conversations should come once a relationship is built up with the family and allowing the family to go at their pace, one professional stated:

*“Each parent… They are all different and they all have different wishes and their different strengths and how they handle the situation. So, I think it’s very much down to the individual and I think it’s the closeness and support and building relationships really, that you can sort of work out how your patients [the parents] are actually dealing with that problem. You can get to know your patients [the parents].” (P6, Community Midwife).*

Another participant referred to the need to look for cues, such as when families start to ask questions that could help to open-up the conversation to approach a discussion around advance care planning; however, this requires professionals to understand and recognise the cues:

*“It’s just that right time, isn’t it? It’s knowing that when somebody says something to you, you can respond to that, to start that conversation. Its understanding what the cues are to that family being ready to have the conversation… Their body language, their non-verbal cues, their language will tell you whether that’s right… That’s probably one of the biggest key points, isn’t it? For practitioners to understand what the cues are.” (P15, Health Visitor).*

#### Parallel planning

Participants spoke of the need for parallel planning to ensure the best plan for the future care of children. For example, where complications had been detected during the pregnancy participants spoke of the need to have ‘parallel planning’, so different plans were ready for all potential outcomes of the birth.

*“Antenatally is when you need… discussions need to start the moment you, as professionals, have concerns about possible palliative care. You need to mention the possibility that what you’re seeing is… it may lead to the individual dying. And then you need to try and set some sort of parallel planning in process.” (P18, Consultant Neonatologist).*

They went on to describe an example where parallel planning had taken place, thus ensuring if the baby was born alive that no active medical intervention was made, and the focus was on providing supportive care:

*“Planning ahead: what we would do and options, and … what might guide our options as well. So, really the aim was to support the baby as they – basically the baby making their own decision as, to, if they was born alive, not implementing any intensive care procedures but to support them in … as good a life as we could achieve; and if the baby wasn’t born alive, to actually give them [the parents] time with the baby.” (P18, Consultant Neonatologist).*

#### Avoiding a crisis situation

However, some participants stated that paediatric ACP conversations should start as soon as possible, even at the point of diagnosis, as it then it avoids the conversations having to take place at a critical time for the parents saying in the situation that if a child suddenly deteriorated:

*“In my personal opinion, it would be as early as possible because things don’t always go to plan. Sometimes children pass away sooner than we think, so probably easier, well not easier but to have something in place for parents so they don’t have to think at the time last minute.” (P17, Hospice Support Worker).*

Similarly, for children with life-limiting conditions it was recognised as important that the timing for the conversations to start needed to be related to the health of the child and for the professional to be aware of any deterioration, emphasising the need for ongoing review for example:

*“With the children with complex needs who kind of bounce in and out of hospital, again it depends on the family, but we generally look at if, say if a child over the last year has bounced in and out of hospital five times whereas the year before they had only been in once, then again we know those families quite well… so we would be having discussions.” (P12, Bereavement Specialist).*

Another participant supported this view by pointing out that such conversations should ideally not take place in crisis situations when parents are under incredible stress:

*“I think in an ideal world it is better to be done when both family and the child is in a more stable situation rather than by the bedside in intensive care when poor family’s feelings are all up the wall, and you know, the child is extremely sick in front on their eyes.” (P21, Hospice Nurse).*

This was reiterated by another professional who referred to staff being relieved when the conversations took place in a calmer setting, particularly as this meant not having to take the parents away from their child at critical times:

*“I think you can see the relief on some professionals’ faces, so they don’t have to ask those awkward questions that in times of intense emotion maybe because the child has become extremely unwell, or for whatever reason, whatever the child is demonstrating at the time, they’re then having to draw them away from their child to ask them specific things.” (P19*, *Palliative Care Nurse Specialist).*

### Supporting effective conversations around advance care planning

#### Where to have the conversation

A vitally important factor in having the conversations centres on where they occur. Good practice was to consider the environment within which the conversation was to take place; one professional stressed:

*“There’s never going to be a nice way to say bad news, but you do have control of environment, you do have control of where you speak to people.” (P14, Hospice Practitioner).*

Another professional described how some families prefer to have conversations in a quieter environment, away from the child in hospital, or another location such as the home. Furthermore, it was highlighted that starting ACP conversations can be facilitated by using photographs of the child. One participant referred to how seeing a photograph helped her to connect with the Mum:

*“I always think it’s lovely to talk to families around pictures because sometimes … they’d rather be in a quieter room or in a room away from that child when we’re having different conversations. Sometimes I meet families where I’ve not met the child… So, for example, that first time I met Mum, I hadn’t seen [child], and I’d said to her, ‘Have you got any pictures?’ and she showed me some pictures on her phone… It’s really important for me to be able to connect with them and it’s really lovely for [parents] to share things with me.” (P15, Health Visitor).*

#### Flexible Planning of the Advance Care Planning Conversations

Timing was also important in starting ACP conversations as soon as possible to allow for a more flexible approach to the conversation, allowing a staged approach. An example was given to illustrate this and how it was done in a conversational style rather than a tick box approach:

*“We didn’t do advance care planning in one sitting, we did it in about three or four sittings so we covered different sections in the advance care plan, you know, at different times; and then we put the plan together and then gave it to the parents ‘ to have a read of” ----“It didn’t feel like you were sitting there with a tick box exercise going through different things; it was more of a conversation that we made sure we covered the elements of the advance care plan in the conversation so we could go back then and document that and write something that looked like an advance care plan to actually share with them, and then the rest of the professionals.” (P13, Senior Hospice Nurse).*

The need to slowly have the conversations and building up over time to allow the news to be absorbed was also reported by several health care practitioners. One professional referred to having conversations over two weeks saying:

*“You let the family set the timeline really ‘cause some families will take a week to get used to the news, and some families will take five minutes to make their mind up about what they want to happen… I can think of having conversations over two weeks with families… twice a day to end up at a point where you’re both agreed that palliation’s the right way forward.” (P18, Consultant Neonatologist).*

#### Introducing the conversation

How to introduce the conversation was an important consideration for the participants. One example was given of how they approach families to initiate conversations around advance care planning when a child is showing signs of deterioration:

*“We can talk about “Oh that must have been really hard” and they would say “Oh it was; they were talking about maybe having to turn the vent off” or whatever… We can use that as a way in to say “Well, you know what, just in case… do you want to sit somewhere and have some conversations about what you want to happen, when that time does happen?”… So, it can just be very simply starting off with some wishes and then they develop.” (P10, Hospice Nurse).*

Another example was given where there had been multiple health care professionals involved in the care of the child, which led to the family feeling they constantly had to repeat the same information each time they met a new professional. This situation enabled the health care professionals to introduce the conversation around advance care planning and the ACP documentation by advocating its value in having a document with everything contained in it to alleviate this issue for parents. One health care professional described the conversation saying:

*“Advance care planning is really important because by actually doing advance care planning we can document what you’ve heard, you know, what your wishes are. And, actually, every time you see a new doctor you can hand them the advance care plan and say, please read this before you come and ask any other questions. So, it was quite handy in that respect for advocating the whole advance care planning process.” (P13, Hospice Nurse).*

This value of this was also reported by other professionals as a means to allow the families to hand over the document and avoid having repeated upsetting conversations.

#### The value of advanced care planning and ACP documentation for families

In addition to saving families from needing to have repeatedly difficult conversation about their child and their wishes, there were other reported practical and emotional advantages of the ACP conversation and documentation. One professional referred to the importance of the family always having a copy of the ACP documentation with them, in case they were, for example, at a grandparent’s home or other venue and needed to call an ambulance. This was reported as being routine practice with the health care professional stating:

*“We actively encourage families to carry a copy of their advance care plan, so that irrespective of if they need an ambulance (where they call it); they have documentation in their possession to be able to demonstrate family’s wishes.” (P19, Palliative Care Nurse Specialist).*

Participants commented on the emotional value of the ACP conversation process for the family. Although it was recognised as a painful experience that could open-up a wealth of emotions and feelings, one health care professional stated that some families express some relief that it has been done:

*“Often, we’re asking to open a Pandora’s box of their emotions and their feelings, and if they want to explore those innermost feelings, and put down on paper the wishes for their child, and whilst that is very emotive and challenging, families often then express the relief that they have done it, and it can be parked as such.” (P19, Palliative Care Nurse Specialist).*

Whether the relief is due to the process of the families verbalising their feelings and potentially facing the fear of the impending death, or the opportunity to be listened to and to plan the best they can for their child is unclear.

## Discussion

This study has explored health care professionals’ views and experiences of paediatric ACP practices and has shown the overarching value of both the process and the documentation. Interestingly, two key areas for practice surrounding ACP conversations were found: identifying the right time to start the conversation with the families; and how to support effective conversations.

There was a mixed response concerning when to start the conversations. With some participants arguing for it to happen as soon as possible, others felt it was better to wait until the parents were ready, and some felt it should wait until they had built up a relationship with the parents. This corroborates the findings from other studies where the readiness of the families to have the conversations was reported [14–16].

Avoiding having the conversation at an emergency or in a crisis was widely reported, which substantiates previous findings [16]. Furthermore, it was suggested there was a need for regular review by the health care team for children with long-term life-limiting illnesses, in order to monitor for deterioration. Despite the overwhelming consensus to avoid starting the ACP conversation in a crisis situation, it was interesting that participants viewed waiting for the family to be ready as their key indicator. One participant referring specifically to ‘picking up the cues’ that a family was ready for the conversations to start. What is unclear from this study is what the cues are and, more so, did all the staff use the same cues, and how did they come to the conclusion that the family were ready to start the conversation? Further research is clearly required to explore this decision-making process. Interestingly in this study, reference was made to building up a relationship with the family before starting ACP conversations. However, it is unclear as to what the participants actually meant by that as they did not verbalise this in a concrete way. It is also uncertain whether the working shift systems operating in the UK health care settings had an impact on their ability to build a relationship with families and contributes to the time delay which can result in a crisis situation developing.

The example of forward and parallel planning for babies who have been found to have some abnormality pre-birth was noted, with a ‘plan for the best, plan for the worst’ approach. This seems to be something that the staff were very familiar with and they spoke of examples where it had worked well. It would be interesting to explore in a larger study whether staff are more comfortable having the ACP conversations before the baby is born, where there are clear timelines to follow, rather than after the birth. This could perhaps be due to the potential hope that still may exist, or due to the education and training that the staff have had. Further research is required to look at this approach to identify lessons to share with the paediatric services, which will support the Perinatal Pathway for Babies with Palliative Care Needs launched in 2017 in the UK [29].

The second key finding from the study relates to supporting good ACP conversations; which included the importance of the setting when having the conversations. The importance of the environment when supporting the families of palliative care patients, when having sensitive conversations, has been previously identified, though it is often neglected, with evidence to suggest that poor environments can increase distress [30]. The need to have a flexible approach to planning over time was highlighted by participants, along with suggestions of how to introduce the topic of ACP drawing upon examples. Suggestions included how it was introduced and developed following a medical crisis where the child survived, as well as ‘advocating the value’ of the ACP documentation to avoid families having repeated difficult conversations. This latter point of ‘selling’ ACP and documentation has been noted in previous research [18], but more in relation to advocating the benefits of having a holistic plan and dismissing the view that the ACP documentation was only about withdrawing potential resuscitation. The practical value of the documentation, where children are being cared for in the community, is noted, so in the event of a rapid deterioration leading to a crisis situation the pre-hospital paramedic staff can see the child and family’s wishes. This is particularly important, so the child and family’s wishes can be conveyed, but even more so if there is a ‘Do Not Attempt Cardio Pulmonary Resuscitation’ order (DNACPR) in place. In the UK, all patients with a DNACPR in place are flagged via electronic referral and information systems that share information with key stakeholders, including the paramedic service, to their home address. If the child was visiting, for example, their grandparents’ home and were not at their home address, active medical intervention could result if the documentation was not to hand [31].

### Limitations of the study

Our recruitment only included professionals who had been directly involved in the end-of-life care of children during the specified timeframe. Therefore, a broader sample considering experience and training levels, especially in advanced communication skills, across wider settings would be valuable. However, the study does include staff from different clinical settings including, hospitals, hospice and community teams from a large geographical area which has provided important and valuable insights into advance care planning in the region.

### Further research

Further research is undoubtedly required, particularly around how the staff come to a decision that a family is ready to start the advance care planning discussions, and which staff felt the timing was right e.g. medical or nursing staff or a combination. A longitudinal observational study would be valuable but challenging.

### Recommendations

1. Clear policy guidance for Health Care Professionals about when to begin the decision-making process surrounding when to start ACP conversations.
2. Education for all Health Care Professionals regarding ACP, including advanced communication skills.

## Conclusion

The study has shown that the timing of when to start the ACP conversations remains an issue for health care professionals. There was a mixture of opinions with some participants feeling that it was to be led by the families and started when a relationship was built up. Conversely, there were others who suggested it should start as soon as possible, therefore avoiding conversation during crisis situations. Examples of parallel planning as part of the standard care and ACP process, which worked well for pregnant mothers, have been reported, supporting the guidance for perinatal pathway for babies with palliative care needs [29]. The study also reported on suggestions to help the process of the ACP. The value of conducting the planning process in stages and considering the environment where the conversation is held being important. These factors were identified as helping the process both for the health care professional but also for families. Clearly timely ACP, with involvement of the appropriate team from specialist palliative care or a children’s hospice for parallel planning, suggest that this will avoid families having difficult conversations at a crisis time. Good ACP is essential for professionals to support the wishes of the child and family, whilst facilitating coordinated care, which will ultimately lead to the best person-centred care for the child and experience for the family.

## Abbreviations

ACP: Advance Care Planning
NHS: National Health Service
NICE: National Institute of Health and Care Excellence
UK: United Kingdom
US: United States of America
